# Sodium Is Not Required for Chloride Efflux via Chloride/Bicarbonate Exchanger from Rat Thymic Lymphocytes

**DOI:** 10.1155/2014/569650

**Published:** 2014-06-09

**Authors:** Donatas Stakišaitis, Vaidevutis Meilus, Alfonsas Juška, Paulius Matusevičius, Janina Didžiapetrienė

**Affiliations:** ^1^Institute of Oncology, Vilnius University, Baublio Street 3b, 08660 Vilnius, Lithuania; ^2^Mykolas Romeris University, Ateities Street 21, 08303 Vilnius, Lithuania; ^3^Lithuanian University of Health Sciences, Veterinary Academy, Mickevičiaus Street 9, 44307 Kaunas, Lithuania

## Abstract

Sodium-dependent Cl^−^/HCO_3_^−^ exchanger acts as a chloride (Cl^−^) efflux in lymphocytes. Its functional characterization had been described when Cl^−^ efflux was measured upon substituting extracellular sodium (Na^+^) by N-methyl-D-glucamine (NMDG). For Na^+^ and Cl^−^ substitution, we have used D-mannitol or NMDG. Thymocytes of male Wistar rats aged 7–9 weeks were used and intracellular Cl^−^ was measured by spectrofluorimetry using MQAE dye in bicarbonate buffers. Chloride efflux was measured in a Cl^−^-free buffer (Cl^−^ substituted with isethionate acid) and in Na^+^ and Cl^−^-free buffer with D-mannitol or with NMDG. The data have shown that Cl^−^ efflux is mediated in the absence of Na^+^ in a solution containing D-mannitol and is inhibited by H_2_DIDS. Mathematical modelling has shown that Cl^−^ efflux mathematical model parameters (relative membrane permeability, relative rate of exchanger transition, and exchanger efficacy) were the same in control and in the medium in which Na^+^ had been substituted by D-mannitol. The net Cl^−^ efflux was completely blocked in the NMDG buffer. The same blockage of Cl^−^ efflux was caused by H_2_DIDS. The study results allow concluding that Na^+^ is not required for Cl^−^ efflux via Cl^−^/HCO_3_^−^ exchanger. NMDG in buffers cannot be used for substituting Na^+^ because NMDG inhibits the exchanger.

## 1. Introduction

**Figure 1:**
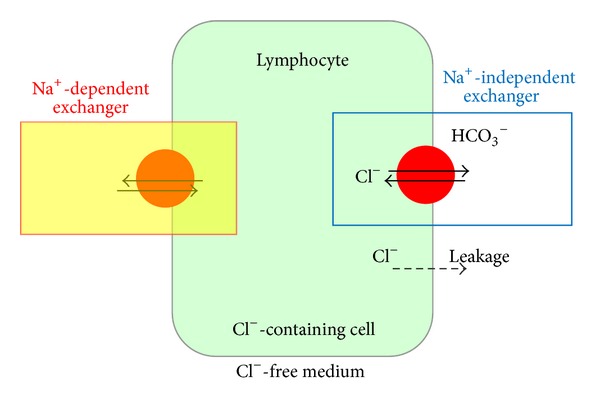
Schematic representation of a thymocyte contained in chloride-free and either sodium* containing* or sodium-*free* medium and pathways and mechanisms of chloride fluxes. Only the pathways and mechanisms of the main concern considered relevant for the present investigation are depicted here. The continuous arrows symbolize the controlled ion flux and the broken one, the uncontrolled flux (leakage), of the ions from the cell. Chloride fluxes (and its efflux) are believed to depend on sodium presence in the medium, that is, on the exchangers of both types (Na^+^-dependent and Na^+^-independent) depicted on both sides of the cell. No contribution of the sodium-dependent exchanger to chloride efflux from rat thymocytes could be observed in this investigation.

Lymphocyte intracellular chloride ([Cl^−^]_*i*_) is regulated by the relative activities of plasma membrane chloride (Cl^−^) influx and Cl^−^ efflux pathways [1]. Literature data show that rat thymocytes (thymic lymphocytes) possess Na-dependent and Na-independent Cl^−^/HCO_3_^−^ exchangers ([1, 2]; Figure 1). So far, two classes of Cl^−^/HCO_3_^−^ exchangers have been described in mammalian cells. The first—the band 3 family of Cl^−^/HCO_3_^−^ exchanger (AE1, AE2, and AE3 isoforms)—is Na-independent [3]. It acts as a Cl^−^ influx mechanism (the intracellular HCO_3_^−^ exchanger for extracellular Cl^−^) [4, 5]. The second exchanger has been identified as a Na-dependent Cl^−^/HCO_3_^−^ exchanger acting as a Cl^−^ efflux mechanism [1, 2, 4]. The description of functional characterization of Na-dependent Cl^−^/HCO_3_^−^ exchangers in lymphocytes has been limited when evaluating fluorimetric intracellular pH change measurements [2]. Later, the same method was used while repeatedly evaluating fluorimetric [Cl^−^]_*i*_ data [1] in the way the Na-dependent Cl^−^/HCO_3_^−^ exchanger was described after cell Cl^−^ efflux had been measured upon substituting extracellular Na^+^ by N-methyl-D-glucamine (NMDG).

In the present study, we examined Cl^−^ efflux by using the fluorimetric Cl^−^ dye N(ethoxycarbonylmethyl)-6-methoxyquinolinium bromide (MQAE) to determine rat thymocyte [Cl^−^]_*i*_ changes during acute exposure to Cl^−^-free media. For Na^+^ and Cl^−^ substitution, we used D-mannitol or NMDG. We show that Cl^−^ efflux is mediated in the absence of Na^+^ in a solution containing D-mannitol and is totally inhibited by H_2_DIDS.

## 2. Material and Methods

Thymocytes were isolated from the* glandula thymus* of male Wistar rats aged 7–9 weeks. Experiments were performed in compliance with the relevant laws and institutional guidelines. The permissions of the State Food and Veterinary Service of Lithuania to use experimental animals for research were obtained (25/07/2013). All bicarbonate-containing buffers were preequilibrated with 5% CO_2_, 20% O_2_, and 75% N_2_ and were kept at 37°C. The composition of buffers is as follows: a standard 100% Cl^−^ buffer (solution 1), a Cl^−^-free buffer (solution 2), a Na^+^- and Cl^−^-free buffer with D-mannitol (solution 3), and Na^+^- and Cl^−^-free buffer containing N-methyl-D-glucamine (solution 4) (Table 1).

[Cl^−^]_*i*_ was measured by the spectrofluorimetric method using MQAE dye as described [1]. Chloride efflux in control thymocytes was measured (*n* = 8) in a buffer in which Cl^−^ had been substituted with isethionate acid (solution 2), in a Na^+^- and Cl^−^-free buffer containing D-mannitol (*n* = 8; solution 3), in thymocytes pretreated with 125 *μ*M H_2_DIDS in a Na^+^- and Cl^−^-free buffer containing D-mannitol (*n* = 4) and in a buffer containing NMDG (*n* = 4; solution 4). The buffer isotonicity was calculated according to NaCl equivalents [6]. Fluorescence measurements were performed with a Perkin Elmer 50B spectrofluorometer (excitation wavelength 352 nm and emission wavelength 450 nm). [Cl^−^]_*i*_ was calculated by using a procedure as described [1, 7]. MQAE, H_2_DIDS, and all other chemicals were purchased from Sigma, Sigma-Aldrich, Fluka, AppliChem, BioEKSMA.

For modelling and model fitting, standard software was used. The models are based on the scheme presented in Figure 1. The systems of simple differential equations were solved with* Maple*. Data processing was carried out using* Microsoft Excel* [8].

## 3. Results

### 3.1. General Considerations, Supposed Relationships, and Modeling

The [Cl^−^]_*i*_ level of thymocytes after their acute exposure to different buffers shows no statistically significant difference (*P* > 0.05): 22.6 ± 2.7 mM in solution 2 (see Table 1), 22.1 ± 3.2 in the same solution pretreated with H_2_DIDS, 21.0 ± 2.9 in solution 3, 21.5 ± 2.4 in the same solution pretreated with H_2_DIDS, and 19.7 ± 1.3 mM with NMDG (solution 4). In the presence of Cl^−^ and HCO_3_^−^ in thymocytes, acute exposure of the cells to an isotonic Cl^−^-free solution and Na^+^- and Cl^−^-free solutions with a D-mannitol substitute resulted in a rapid decline of [Cl^−^]_*i*_.

The current understanding of the mechanisms of Cl^−^ efflux from thymocytes is presented in the scheme depicted in Figure 1. The efflux from the cells in which the activity of the exchanger is inhibited has to be assigned to a noncontrollable leakage of chloride anions through the membrane. The rate of the leakage (*F*_leak_), presumably, can be expressed as follows:

(1) $$\begin{array}{c} F_{\text{leak}}=-\alpha y, \end{array}$$

where *α* is relative permeability of cellular membrane and *y* is transmembrane difference of Cl^−^ concentration or just Cl^−^ concentration in the cytoplasm (if the medium is Cl^−^-free). The solution of the above equation is

(2) $$\begin{array}{c} y=y_{0}\exp\left(-\alpha t\right), \end{array}$$

**Figure 2:**
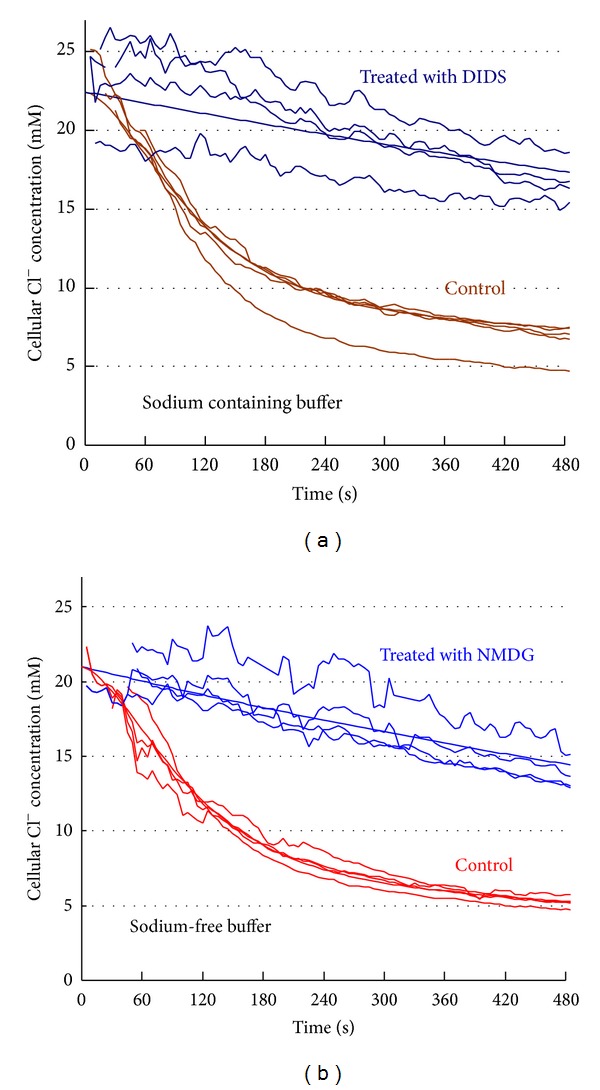
Chloride efflux from thymocytes (treated either with H_2_DIDS (*n* = 4) or with NMDG (*n* = 4)) into chloride-free, bicarbonate-*containing,* and either sodium-*containing* or sodium-*free* medium. The smooth curves correspond to model (2) (noncontrollable chloride efflux) or model (4) (taking into account the contribution of the exchanger) with the parameter values presented in Table 2. The controls were Na isethionate (a) and D-mannitol (b). The data (zigzagged lines) are representative of *n* = 8 (a) and *n* = 8 experiments (b).

*y*_0_ being initial Cl^−^ concentration in the cytoplasm. The model is depicted in Figure 2.

Experimental data of cytoplasmic Cl^−^ concentrations in the absence of Cl^−^/HCO_3_^−^ exchange inhibition (Figure 2) suggest the decline to proceed with the relative rate different from constant. At the beginning it is slow, then fast, and slow again, keeping in mind that the observed decline in Cl^−^ concentration results from both noncontrollable Cl^−^ leakage via the cellular membrane and its efflux via the exchanger. The total efflux rate can be presented as follows [8]:

(3) $$\begin{array}{c} \frac{\text{d}y}{\text{d}t}=-\alpha y-A\frac{\lambda \mu }{\lambda -\mu }\left(\exp\left(-\mu t\right)-\exp\left(-\lambda t\right)\right), \end{array}$$

where *A* symbolizes the efficacy of the exchanger and *λ* and *μ* stand for relative rates of rise and decline of its activity, the parameters *λ* and *μ* being interchangeable. The solution of the above equation is

(4) y=y0exp⁡(−αt)−Aexp⁡(−αt)λμ×((μ−α)exp⁡(−(λ−α)t)(λ−α)(μ−α)(λ−μ)  −(λ−α)exp⁡(−(μ−α)t)+(λ−μ)(λ−α)(μ−α)(λ−μ)).

### 3.2. Comparison of Models and Experimental Data

The data together with the models are presented in Figure 2. The models seem quite acceptable suggesting the plausibility of the initial assumptions; together with the parameters contained in Table 2 they are discussed in detail in the next section.

## 4. Discussion

It is commonly accepted that in rat thymocytes the intracellular Cl^−^ level is regulated by Na-independent and Na-dependent Cl^−^/HCO_3_^−^ exchangers and Na-K-2Cl cotransporter [1, 2]. Two classes of Cl^−^/HCO_3_^−^ exchangers related to [Cl^−^]_*i*_ level regulation were identified in mammalian cells: band 3 family (AE1, AE2, and AE3) was found to be Na-independent [3]. It normally acts as a Cl^−^ influx mechanism [2, 9]. Lymphocytes express the AE2 isoform which shows a lower affinity for DIDS than does AE1 [4, 9, 10]. The Cl^−^/HCO_3_^−^ exchanger, which was described as a Na-dependent Cl^−^/HCO_3_^−^ one, acts as a Cl^−^ efflux mechanism in experiments of external Cl^−^ removal [2, 11]. The Na-dependent Cl^−^/HCO_3_^−^ exchanger in thymocytes was described when it was evaluated by measuring intracellular pH changes [2]. Later, this exchanger was evaluated by measuring [Cl^−^]_*i*_ in the experimental conditions identical to those reported earlier [1]. Evidence for the existence of a Na-dependent Cl^−^/HCO_3_^−^ exchanger comes from studies examining the effects of DIDS and Na^+^ removal on net Cl^−^ efflux, in which Na^+^ and Cl^−^ were substituted by NMDG. Such experimental data show that the net Cl^−^ efflux is completely blocked by the acute removal of Na^+^ from the external medium, and this fact led to the conclusion that Cl^−^/HCO_3_^−^ exchange was due to a Na-dependent mechanism. The nature of the “blockade” mechanism is not clear, nor has the Na-dependent Cl^−^/HCO_3_^−^ exchanger been isolated and cloned to date.

The study was undertaken to examine the Na^+^ involved in Cl^−^ efflux from rat thymocytes, using another Cl^−^ and Na^*+*^ substitute D-mannitol. Examination of the Cl^−^ efflux pathway was performed by acutely exposing cells to Na-Cl^−^-free media and determining changes in the [Cl^−^]_*i*_ level. The basal [Cl^−^]_*i*_ level in the study of thymocytes was similar to [Cl^−^]_*i*_ levels as found by others for different cell types such as rat thymocytes [1], vascular smooth muscle cells [7, 12], and astrocytes [13].

In the presence of Cl^−^ and HCO_3_^−^ in the thymocytes, acute exposure of the cells to an isotonic Cl^−^-free solution and Cl^−^- and Na^+^-free solutions with a D-mannitol substitute resulted in a rapid decline of [Cl^−^]_*i*_. The parameters of the mathematical model (relative membrane permeability, relative rate of exchanger transition, and exchanger efficacy) show that Cl^−^ efflux in a bicarbonate buffer in which Cl^−^ is substituted with Na isethionate is the same as in the Na^+^-Cl^−^-free buffer in which these ions are substituted isotonically with D-mannitol.

We stress that the buffer for experiments examining Na-dependent Cl^−^ efflux where Na^+^ and Cl^−^ are substituted by NMDG cannot be used for evaluating Cl^−^ efflux. The NMDG properties listed below should be estimated while investigating a Na-dependent Cl^−^/HCO_3_^−^ exchanger. The use of NMDG to substitute monovalent cations requires pH adjustment [14–16]. Following the reconstitution with deionised water, the pH of 185 mM of the NMDG solution was 11.4 due to the alkaline properties of NMDG (its molecule contains a charged methylamine head group responsible for alkalinity), as NMDG has the ionisation equilibrium constant *K*_*b*_ = 3.98 · 10^−5^ [17]. Adjustment of the pH of its solution requires large quantities of acids: 125 mM of H_2_SO_4_ has to be added to adjust the pH of the solution to 7.3. The osmolarity of the solution was 6.2 atm before pH adjustment and 17.4 atm after it (own calculation).

Historically, NMDG has been used for several decades for the substitution of monovalent cations, assuming that it does not cross the cell membrane. However, today our knowledge of NMDG permeability through ion channels has undergone essential changes. NMDG permeation has been reported in ion channels such as ATP-gated P2X [18], epithelial Ca^2+^ channel ECaC [19], glutamate receptor [20], mechanosensitive channels [21], and mutant Na^+^ channels [22]. Some transient receptor potential family cation channels display partial permeability to NMDG [23]. In the absence of K^+^, significant NMDG currents were recorded in human kidney cells expressing Kv3.1/Kv3.2b and Kv1.5 R487Y/V channels. Inward currents were much stronger because of the blockade of the outward currents by intracellular Mg^2+^, resulting in a strong inward rectification [16]. NMDG rapidly blocks Ca^2+^-activated K^+^ channels from the inside of the membrane [24]. Extracellular NMDG causes a partial block of outward currents in the TRPC3 member of the transient receptor potential family cation channels [25], and the intracellular NMDG modifies the properties of the Ca^2+^ L-type channel in guinea pig cardiac myocytes by increasing the overall duration of the Ca-dependent slow action potential 6-fold at 0 mV [26]. NMDG increases the intracellular Ca^2+^ and K^+^ in Ehrlich Lettre ascite cells, changing the intracellular pH [27].

When using NMDG for Na^+^ substitution, the solution hypertonicity, alkaline features of the substitute, and an additional high concentration of anions appearing after buffer adjustment with acids could change the functioning of the exchanger. AE2 studies have also shown that it is capable of transporting a number of different anions working in a number of different modes of exchange such as Cl^−^/Cl^−^ and sulfate/chloride [28]. Band 3 protein may also be able to exchange Cl^−^/OH^−^ [29]; besides, it has been shown to transport sulphate [30, 31] and phosphate anions [32].

**Figure 3:**
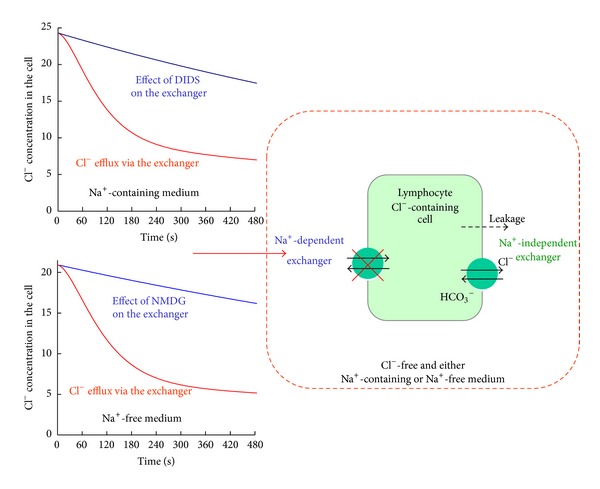
The summarized results of the Cl^−^ efflux study.

The study data show that there is no Cl^−^ efflux from thymocytes in a bicarbonate buffer in which Na^+^ and Cl^−^ are substituted by NMDG. However, Cl^−^ efflux through Cl^−^/HCO_3_^−^ exchanger occurs when the neutral substitute D-mannitol is used instead of NMDG. This means that extracellular Na^+^ is not required for Cl^−^ efflux in rat thymocytes. This could be in agreement with studies indicating that in experimental conditions of external Cl^−^ removal the AE2 antiporter can be reversed; that is, it works at both sides of a lymphocyte membrane [4, 33]. Pretreatment of cells with DIDS before exposure to a Cl^−^-free solution inhibited the decline in [Cl^−^]_*i*_, suggesting that the Cl^−^/HCO_3_^−^ exchange mechanism is responsible for Cl^−^ efflux in these cells. The stilbene compound (DIDS, SITS) binding site is located on the outer surface of the membrane rather than buried within the pocket formed by the tertiary complex of the protein [34, 35]. No contribution of Na dependence of Cl^−^/HCO_3_^−^ exchanger has been observed in the study. The main data of the study are summarized in Figure 3.

Hence, it follows that the presence or absence of Na^+^ in the extracellular medium is not relevant for Cl^−^ efflux from thymocytes, implying that Cl^−^ efflux in experimental conditions is related to Cl^−^/HCO_3_^−^ exchanger (band 3) in thymocytes. Sodium substitution with NMDG leads to an inhibition of the Cl^−^/HCO_3_^−^ exchanger. The higher estimate of noncontrolled leakage (*α*, see Table 2) suggests an incomplete inhibition at the NMDG concentration used in the study.

## 5. Conclusions

The main results of this work can be summarized as follows:

- Na^+^ is not required for Cl^−^ efflux via Cl^−^/HCO_3_^−^ exchanger from rat thymocytes;
- no Na-dependent Cl^−^/HCO_3_^−^ exchanger is present in rat thymocytes;
- NMDG leads to exchanger inhibition.

## Figures and Tables

**Table 1:** Solution compositions (mM).

Ingredient	Solution 1 (100% Cl^−^)	Solution 2 (Cl^−^-free)	Solution 3 (Cl^−^- and Na^+^-free with D-mannitol)	Solution 4 (Cl^−^- and Na^+^-free with NMDG)
Glucose	5	5	5	5
HEPES	5	5	5	5
MgSO_4_	0.8	0.8	0.8	0.8
NaH_2_PO_4_	1	1	0	0
KH_2_PO_4_	0	0	1	1
Ca acetate	1.8	1.8	0	0
NaCl	96	0	0	0
Na isethionate	16.4	117.3	0	0
D-mannitol	0	0	185	0
KCl	5.3	0	0	0
N-methyl-D-glucamine	0	0	0	185
K gluconate	0	0	4.3	0
KHCO_3_	0	5.3	0	4.3
NaHCO_3_	22	16.7	0	0
Choline HCO_3_	0	0	22	22

**Table 2:** Conditions of the experiments and model parameters estimated by fitting (2) and (4) to experimental data.

Parameter	Notation	Estimate	
		Na^+^-containing medium (solution 2)	Na^+^-free medium (solution 3)
Initial chloride concentration in cytoplasm, mM	*y*_cytoplasm_	22.6	21.0
Relative membrane permeability, s^−1^	*α*	0.000533	0.000774
A rate constant of the exchanger transition, s^−1^	*λ*	0.000547	0.00547
Another rate constant of the exchanger transition, s^−1^	*μ*	0.0169	0.0169
Exchanger efficacy, mM	*A*	12.1	12.2
